# Computational Design of Single-Peptide Nanocages with Nanoparticle Templating

**DOI:** 10.3390/molecules27041237

**Published:** 2022-02-12

**Authors:** José A. Villegas, Nairiti J. Sinha, Naozumi Teramoto, Christopher D. Von Bargen, Darrin J. Pochan, Jeffery G. Saven

**Affiliations:** 1 Department of Chemistry, University of Pennsylvania, Philadelphia, PA 19104, USA; josev@uic.edu (J.A.V.); vonc@sas.upenn.edu (C.D.V.B.); 2 Department of Materials Science and Engineering, University of Delaware, Newark, DE 19716, USA; sinha@udel.edu (N.J.S.); teramoto@udel.edu (N.T.)

**Keywords:** peptides, self-assembly, molecular cages, computational design, biomaterials

## Abstract

Protein complexes perform a diversity of functions in natural biological systems. While computational protein design has enabled the development of symmetric protein complexes with spherical shapes and hollow interiors, the individual subunits often comprise large proteins. Peptides have also been applied to self-assembly, and it is of interest to explore such short sequences as building blocks of large, designed complexes. Coiled-coil peptides are promising subunits as they have a symmetric structure that can undergo further assembly. Here, an α-helical 29-residue peptide that forms a tetrameric coiled coil was computationally designed to assemble into a spherical cage that is approximately 9 nm in diameter and presents an interior cavity. The assembly comprises 48 copies of the designed peptide sequence. The design strategy allowed breaking the side chain conformational symmetry within the peptide dimer that formed the building block (asymmetric unit) of the cage. Dynamic light scattering (DLS) and transmission electron microscopy (TEM) techniques showed that one of the seven designed peptide candidates assembled into individual nanocages of the size and shape. The stability of assembled nanocages was found to be sensitive to the assembly pathway and final solution conditions (pH and ionic strength). The nanocages templated the growth of size-specific Au nanoparticles. The computational design serves to illustrate the possibility of designing target assemblies with pre-determined specific dimensions using short, modular coiled-coil forming peptide sequences.

## 1. Introduction

The design of protein and peptide cages holds potential for a variety of biomaterial and medical applications [1,2,3,4,5,6]. For example, protein nanocages have been used for drug encapsulation [7,8], enzyme encapsulation [9], and for constructing size-constrained nano-reactors [10,11,12]. The cage exterior can be designed to tune solubility, porosity, and the diffusion rates of molecular species in and out of the cage [13,14]. Furthermore, the cage exterior can be decorated to impart functions such as target recognition, biosensing, and target delivery [15]. Computational protein design has been used extensively in the design of novel protein-based symmetric structures [16,17,18,19,20], but tunability of the interior cage volume has been limited by two factors: the use of natural proteins as building blocks and the need to arrange the subunits in strictly symmetrical arrangements [21].

Rational and computational approaches have been previously used in the design of highly symmetric protein assemblies, such as homo-oligomeric and hetero-oligomeric icosahedral cages derived from naturally occurring proteins [16,17,18,19,22,23,24]. Natural proteins are often large, complex and require heterologous expression [25]. The folding of such constructs can often be irreversible. Such proteins may also be sensitive to modification, which can lead to unpredictable loss in structure and/or function. In order to provide alternatives, there have been efforts to build artificial nanocages using α-helical peptides [26]. The symmetry of structure and periodicity in sequence can be leveraged in the design of sequences, and nonbiological modifications are readily synthetically incorporated in a site-specific manner [27]. Moreover, using short peptide sequences probes the extent to which self-assembly and complexity can be encoded in relatively short amino acid sequences. For example, Jerala and co-workers used single peptide chains that folded into homodimeric coiled coils to build self-assembling monodisperse tetrahedral and triangular bipyramid cages of ~6.9 nm ~12.4 nm, respectably [28,29,30]. In another remarkable attempt, a stable ~16 nm self-assembling nanocage was constructed by Burkhard and co-workers using a single 36 amino acid polypeptide sequence as the molecular building block. The subunits contained two coiled-coil forming peptide domains with different oligomerization states, i.e., five and three, joined by a short linker [31,32]. Fletcher et al. built stochastic, self-assembling cage (SAGE) particles that were approximately 100 nm in size [33], in which a 24-residue homotrimeric coiled-coil forming peptide was fused with a 24-residue homodimeric coiled-coil forming peptide via di-sulfide linkers. The fused peptides self-assembled in solution to form a honeycomb nano-structure with an unforeseen curvature that resulted in the formation of porous enclosed structures. While these recent attempts are encouraging, the need remains for developing a peptide-based tunable nanocage design and assembly strategy.

The other common limitation on size tunability in computational design is the use of strictly symmetrical arrangements, since the number of subunits in the assembly is restricted by the chosen point-group [34]. Point-group symmetry is a common feature of protein assemblies, as it places all subunits in identical local environments [35]. In a strictly symmetrical assembly, every subunit is positioned identically relative to its neighbors and interacts by forming the exact same interfaces. However, protein surfaces are highly dynamic and can sample multiple surface topologies. This enables the formation of protein complexes in which identical molecules interact in distinct ways depending on their position within the assembly [36]. Symmetry breaking allows for the placement of *nm* subunits within the assembly, where *n* is the number of distinct interfaces and *m* is the number of symmetry-related elements in the point group. This breaking of symmetry is commonly utilized by viruses to construct large capsids from a single protein molecule. Woolfson and co-workers observed that peptide coiled coils can collapse to form low-symmetry assemblies, in which identical chains in a homomeric structure adopt different conformations [37], and that symmetrical and non-symmetrical structures can co-exist in equilibrium [38]. However, artificial spherical synthetic peptide assemblies that exhibit symmetry breaking have often only been obtained serendipitously or through screening methods [39,40]. Recently, the principles of symmetry breaking are beginning to be elucidated from the self-assembly of small synthetic foldamer subunits [41,42,43]. Such symmetry concerns are relevant for the design of small peptides that oligomerize to form complex assemblies.

Herein, we focus on the computational design of a functional nanocage comprising a single, short peptide sequence (less than 30 residues). We targeted an assembly that should be clearly evident in experimental studies, where the function in this case is the templated growth of Au nanoparticles having a size determined by the cavity of the nanocage. A peptide homodimer was chosen as the building block of the assembly. A probabilistic approach was used to calculate the probabilities of amino acids and their side-chain conformers at each variable site in the sequence. Amino acid probabilities were constrained so that equivalent residue positions on different chains have identical amino acid probabilities but may have distinct side-chain conformational states. In this manner, we identified a single sequence compatible with two distinct local environments in the dimer and within an octahedral arrangement. The resulting 29-residue peptide sequence was computationally designed to form an 8.6 nm nanocage, in which the cage comprises 48 chains each with the same sequence. Seven such designed sequences were experimentally realized and characterized. One of the sequences exhibited behavior consistent with the targeted nanocage self-assembly. Dynamic light scattering (DLS) and transmission electron microscopy (TEM) together confirmed that the peptides formed spherical assemblies of target size in the solution, thus corroborating the success of the computational design protocol. The cages templated the growth of Au nanoparticles upon the well-known reduction in auric salt with borohydride, yielding nanoparticles of 8 nm in diameter.

## 2. Materials and Methods

### 2.1. Computational Design of Self-Assembling Peptides

An atomic coordinate file for GCN4-pV [44] was obtained from entry in the Protein Data Bank (PDB 2B22). The structure was modified to provide an N-terminal acetyl and a C-terminal amide; each was added to the structure using PyMol [45]. The GCN4-pV structure contains one α-helix in the asymmetric unit, and the antiparallel tetramer can be generated from the symmetry operations of the I4_1_22 crystal space group. The structure was used to obtain one half of the native tetrameric coiled coil, corresponding to the antiparallel dimer. This dimer formed the building block of the tetramer and larger assembly. The tetrameric structure was centered at the origin. The nanocage structure was generated by considering translations and rotations along a *C*_2_ symmetry axis of the tetrameric bundle (x = y at z = 0) and calculating the atomic coordinates of symmetry-related elements within *O*_h_. The 24 symmetry operations for the *O*_h_ point group were obtained from the F432 space group table in the International Tables for Crystallography [46]. In the F432 space group, the x = y line at z = 0 is one of the *C*_2_ axes in the set of transformations used to generate the *O*_h_ symmetry. The symmetry-related element generated by this operation from the dimeric asymmetric unit results in the formation of the full tetrameric coiled coil as well as the octahedral cage.

**Figure 1:**
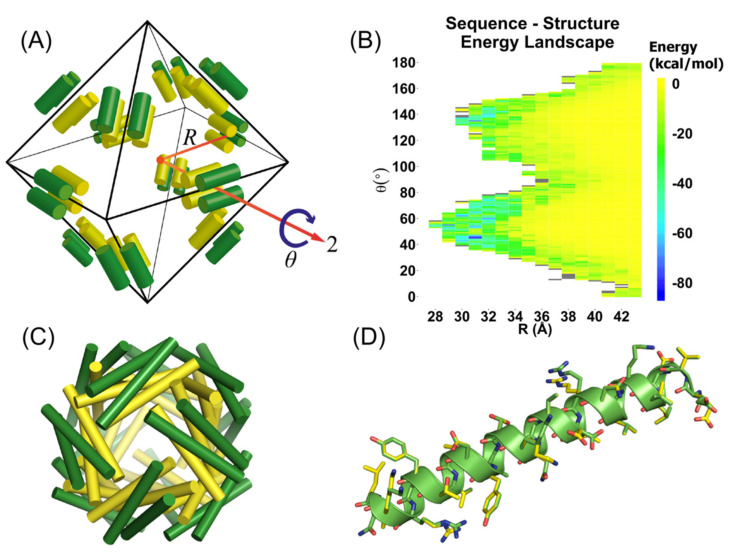
Computational design of peptidic coiled-coil cages: (**A**) asymmetric unit (dimer) contains one helix in green and one helix in yellow. This dimer is rotated by an angle of *θ* and translated by distance *R* along the axis vector. The cage is generated by applying the symmetry operations of the *O*_h_ point group. The two helices in the asymmetric unit are located in distinct local environments, where the yellow helices make up the inner layer of the cage, and the green helices make up the outer layer of the cage; (**B**) sequence–structure landscape; average energy over sequences and all allowed side-chain conformations as a function of *R* and *θ*; (**C**) structure of peptide cage at *R* = 30 Å and *θ* = 44°; and (**D**) most probable structure of the two chains in the asymmetric unit after sequence design imposing sequence symmetry constraints. Not all amino acids have identical side-chain conformations.

The radius *R* is defined as the displacement along the *C*_2_ axis, while *θ* is defined as the rotation about this axis (Figure 1A). Values of *R* varied from 28 Å to 43 Å at 0.1 Å intervals and values of θ were varied from 0° to 180° at 1° intervals. Virtual copies of the asymmetric unit (dimer) were created in an octahedral arrangement and were generated using the 24 symmetry transformation matrices of the F432 crystal space group. For each such nanocage geometry, the properties of a sequence ensemble with the degrees of freedom shown in Table S1 were considered. As in the heptad positions of the parent GCN4-pV sequence, some residue identities were retained: valine residues at the *d* and *e* positions, leucine residues at the *a* positions, and asparagine at position 16 (*e* position). The resulting ensembles allowed a total of 4502 unique identity-rotamer (monomer) states. For each structure, the site-specific probabilities of the residues and their side-chain conformations were determined using an entropy-based approach subject to constraints on the sequences as previously described [18,23,47,48,49,50,51,52]. The calculation yields the site-specific probabilities and average energy over the sequence ensemble. For a given configuration of the nanocage, monomer states having atomic overlaps (high energy interactions) with backbone atoms of any subunit or with side-chains at symmetrically equivalent positions were removed prior to the probability calculation. 

For each candidate nanocage structure, the calculations yield an effective internal energy landscape as a function of the geometric parameters of the nanocage (Figure 1B). The 32 nanocage structures on the landscape with the lowest internal energies were chosen for sequence design calculations. At each of these points, an ensemble was generated with the same amino acid degrees of freedom indicated in Table S1, but all conformations in the Dunbrack rotamer library were allowed [53]. An additional constraint was included in the entropy-based calculation of the residue probabilities. The building block was a dimer of two chains, here labeled *A* and *B*. At each residue position *i* of each of the chains *A* and *B* within this asymmetric unit, *m_i_* amino acids and *c_mi_* conformations of each amino acid are considered. We let *α* denote the amino acid and *r_n_*(*α*) the *n*-th side chain rotamer state of this amino acid; *m*−1 constraints were imposed to constrain the fact that the amino acid probabilities and equivalent sites on chains *A* and *B* are equal for all amino acids allowed at site *i*. In this manner, the probabilities of amino acids at equivalent sequence positions, $w_{i}^{A}\left(\alpha \right)$ and of chains *A* and *B* are constrained, but the side-chain conformations are not:

$$w_{i}^{A}\left(\alpha \right)=\overset{c_{mi}}{\underset{n}{\sum }}w_{i}^{A}\left(\alpha ,r_{n}\left(\alpha \right)\right)=\overset{c_{mi}}{\underset{n}{\sum }}w_{i}^{B}\left(\alpha ,r_{n}\left(\alpha \right)\right)=w_{i}^{B}\left(\alpha \right)$$

Here, $w_{i}^{A}\left(\alpha ,r_{n}\left(\alpha \right)\right)$ and $w_{i}^{B}\left(\alpha ,r_{n}\left(\alpha \right)\right)$ are the probabilities of amino acid *α* and side-chain conformation *r_n_*(*α*) at position *i* in chains *A* and *B*, respectively. For each equivalent site *i* on chains *A* and *B*, an amino acid was removed from consideration on both chains if it was not tolerated on either chain due to atomic overlap or other high-energy interactions involving all its side chain conformations.

The calculation iteratively proceeded for each structure of the assembly. If at any given site the most probable amino acid was more than twice as probable than the next most probable amino acid, the site was constrained to this most probable amino acid. The determination of the monomer state probabilities using the reduced ensemble was carried out, and the procedure was repeated until no further amino acids were specified. The calculated probabilities were used to select the sequences and conformations for further computational validation. 

All candidate sequences and dimer structures were uploaded to the Proteins, Interfaces, Structures and Assemblies (PISA) server [54], and the assemblies were analyzed. To further assess the likelihood of forming the targeted peptide assemblies in solution, each candidate coiled coil was subjected to a trimeric protein–protein docking calculation using the ClusPro web server [55,56,57,58,59]. A coordinate file of only a single tetrahelical coiled coil was uploaded to the server as the receptor, the multimer docking option was selected, and the number of subunits was specified as three. The program predicted the structure of a trimer of helical bundles using only the tetrahelical coiled coil structure. Only designs that were identified as the target assembly by PISA and ClusPro were considered for experimental validation. PISA defines as stable a biological assembly with a calculated energy of dissociation score greater than 0 kcal/mol. 

The agreement between ClusPro docking prediction and the designed cage models was judged visually. The representative structures of the most populated cluster for each set of coefficients (“balanced”, “electrostatic-favored”, “hydrophobic-favored”, and “VdW+Elec”) were visually compared to the predicted cage assembly to see whether each docked chain in the ClusPro prediction could be assigned to an equivalent chain in the design. The backbone root mean square deviation (RMSD with backbone atom types N, C, CA, and O) between the prediction and the design was calculated. Seven candidates were selected as those having a low internal energy from the sequence calculations, low predicted PISA assembly scores, and an alignment of ClusPro docking structures.

### 2.2. Experimental Methods

#### Peptide Synthesis and Purification

Computationally designed peptide sequences that were selected for experimental investigation were synthesized using standard Fmoc-based solid-phase peptide synthesis protocols on CEM corporation’s Liberty Blue synthesizer [60]. A 0.1 × 10^−3^ mole scale microwave-assisted solid-phase peptide synthesis (MW-SPPS) was performed using side-chain protected Fmoc-amino acids and Rink-Amide resin (200-mesh, 0.53 g/mmol) supplied by ChemPep Inc. (Wellington, FL, USA). Chemicals 2-cyano-2-(hydroxyimino) acetate (OxymaPure^®^) and N, N′-diisopropylcarbodiimide (DIC) were used as the activation mixture and 20% piperidine was utilized for deprotection, all of which were purchased from Sigma Aldrich (St. Louis, MO, USA). All chemicals were dissolved in analytical grade dimethylformamide (Fisher Scientific, Waltham, USA) in quantities recommended by CEM. Deprotection and coupling steps were performed under microwave heating at 90 °C separated by multiple DMF washes. The N-terminus of the final peptidoresin was acetylated using a 10 mL solution containing 20% acetic anhydride, 80% DMF, and 300 μL N, N-Diisopropylethylamine. The resulting acetylated peptidoresin was washed once with dichloromethane (DCM), twice with DMF, and dried under nitrogen gas for at least 30 min. To cleave the peptide off the resin, the peptidoresin was suspended in 10 mL of a cleavage cocktail containing 95% trifluoroacetic acid (TFA), 2.5% Milli-Q water, and 2.5% triisopropylsilane (TIPS) for two hours. The cleaved peptide solution was then separated from the resin through a polypropylene filter and was thrice precipitated in fresh anhydrous ethyl ether (Fisher Scientific), followed by centrifugation and drying under nitrogen gas overnight. The crude peptide was dispersed in fresh water and lyophilized under high vacuum at −85 °C for subsequent purification.

The lyophilized crude peptide was dissolved in an appropriate mixture of Milli-Q water and HPLC grade acetonitrile (Fisher Scientific) and filtered using a 0.2 μm polyethersulfone (PES) syringe filter (Corning^®^). Purification was performed on a semi-preparative scale reverse-phase high-pressure liquid chromatography (RP HPLC) instrument having a BEH130 Prep C18 10 mm column (XBridge, Waters Corporation, Milford, MA, USA). A mixture of Milli-Q water (solvent A) and acetonitrile (solvent B) both with 1 *v*/*v* % formic acid (Fisher) was utilized as the mobile phase, with an elution gradient of 85 *v*/*v* % solvent A to 25 *v*/*v* % solvent A in 60 min. The peptide elution was followed at 280 nm using an ultraviolet–visible detection system (Waters 2489, Waters Corporation). Electron-spray ionization mass spectrometry (SQ detector 2, Water Corporation) was performed to check for the purity of the collected peptide. Finally, the resulting pure peptide solutions were combined, lyophilized and checked for secondary structure using circular dichroism spectroscopy (see Figures S1–S3 in the supplemental information document for peptide purity, molecular weight, and secondary structure characterization, respectively). 

### 2.3. Nanocage Self-Assembly

Purified and lyophilized peptide was dissolved in 6M Guanidine hydrochloride prepared in Milli-Q water to denature the peptide. The concentration of the peptide solution was checked on a UV-Visible spectrophotometer and adjusted to 0.4 mM. Specifically, the absorption by tyrosine at 280 nm was measured and using an extinction coefficient ϵ of 2560 M^−1^ cm^−1^, and the concentration of the peptide was calculated by the Beer–Lambert law. 

For self-assembling the peptides into nanocages, the peptide solution was first diluted to 0.2 mM by the addition of Milli-Q water. The solution was then filtered with a 0.2 micrometer polyethersulfone (PES) syringe filter (Corning^®^) into a 0.5 mL Thermo-Fisher dialysis cassette^®^ with a 2000 Dalton molecular weight cutoff (2kD MWCO). Extensive dialysis was carried out in a step-wise manner against 500 mL dialysis buffer under mild agitation at 20 °C. First, dialysis was carried out against 1 M guanidine hydrochloride, followed by 100 mM and 1 mM guanidine hydrochloride dialysis steps and finally, against Milli-Q water twice, so that the final solution was estimated to contain less than 5 × 10^−9^ M guanidine hydrochloride. Alternatively, the final dialysis steps were performed against target buffer solutions of either 5 mM or 10 mM buffer concentrations, i.e., acetate buffer (pH 4.5), phosphate buffer (pH 7.0), and borate buffer (pH 9.5). All dialysis buffers were prepared fresh in Milli-Q water for optimum buffering at 20 °C and filtered using a 0.2 micrometer PES syringe filter before use.

### 2.4. Transmission Electron Microscopy (TEM)

For the TEM characterization of solutions containing nanocages and incubated gold nanoparticles, 200 mesh copper grids coated with an ultrathin layer (3–4 nm) of carbon (CF200-Cu-UL) were purchased from Electron Microscopy Sciences Inc. For Cryo-TEM, lacey carbon grids were used. The grids were plasma treated by glow discharge (PDC-32G, Harrica Plasma Inc., Ithica, NY, USA) for 30 s. Five microliters of 0.05 × 10^−3^ M peptide nanocage solution was added to the carbon side of the grid. For cast-film images, the solution was allowed to sit for 2 min before blotting the excess liquid using the edge of a dry filter paper. The sample was washed with 3 microliter water and immediately blotted to remove excess peptide and buffer salt. The grid was dried for 15 min under air and used for TEM analysis within an hour of preparation. For Cryo-TEM, the sample grid was prepared using the Vitrobot instrument. Both Cryo and cast-film TEM were performed on a Thermo Scientific™ Talos™ F200C transmission electron microscope equipped with a 4 k × 4 k CMOS camera Ceta 16M. When imaging the nanocage assembly, an accelerating voltage of 120 kV was utilized to enhance contrast—whereas for imaging gold nanoparticles incubated within the cages, an accelerating voltage of 200 kV was employed. All image analyses were performed using ImageJ analysis software [61]. First, using the freehand tracing tool, more than 300 nanocages were selected across multiple TEM images. Using ImageJ’s measure tool, the average Feret diameter and fitted ellipse dimensions of the selections were calculated and averaged. The size distribution was recorded using ImageJ’s Distribution tool.

### 2.5. Dynamic Light Scattering

Dynamic light scattering (DLS) was carried out on Malvern’s Zetasizer Nano ZSP instrument at 20 °C using a 173° back-scatter angle to quantify the hydrodynamic diameter of peptide assemblies after dialysis. Samples were prepared by filtering and sonication before transferring into a clean quartz cuvette. The viscosity and refractive index of the peptide solution was assumed to be the same as that of pure water for calculating the autocorrelation function. Data were collected for 20 s, and results from 10 runs were averaged to calculate the intensity average size-distribution using a CONTIN data fitting software.

### 2.6. Incubation of Gold Inside Peptide Nanocages

A 0.1 × 10^−3^ M peptide solution containing pre-assembled nanocages was utilized to mineralize gold nanoparticles using a protocol reported previously [62]. We used a 200 molar excess of gold (III) chloride hydrate (HAuCl_4_, Sigma, St. Louis, MO, USA) in Milli-Q water which was filtered using a 0.2 micrometer PES syringe filter and was incubated with nanocage solution for 24 h to enable the sequestration of the gold ions into the core of the nanocage. An equimolar sodium borohydride (NaBH_4_, Fisher) solution with HAuCl_4_ was then added to the peptide solution to reduce the Au (III) to Au (0). The resulting reddish-brown solution was centrifuged at 5000 rpm for two minutes and the clear supernatant was evaluated for the formation of gold nanoparticles. 

## 3. Results

### 3.1. Computational Design of Peptide Cages

To design self-assembling peptide nanocages, we targeted an assembly of coiled-coils in a spherical arrangement with octahedral symmetry. We selected the antiparallel tetrameric coiled-coil GCN4-pV [44] as the backbone template upon which to design protein–protein interfaces that would lead to the self-assembly of the nanocages. GCN4-pV is a highly stable tetramer of α-helices, which led us to hypothesize that this structure would tolerate substantial sequence variation. By maintaining the amino acid identities at core positions along the heptad repeat, we sought to ensure that the tetrameric oligomerization state and internal symmetry were maintained. The *D*_2_ symmetric arrangement of the tetramer means that there are three perpendicular *C*_2_ axes, which cannot be mapped simultaneously onto the *O*_h_ point group. As a consequence, the asymmetric unit must be composed of a dimer of α-helices with one internal *C*_2_ axis. A second *C*_2_ axis can be mapped onto one of the *C*_2_ axes of the *O*_h_ point group. Applying all symmetry operations results in the formation of a spherical arrangement of 12 tetrameric coiled-coil subunits constructed from 24 asymmetric (dimer) units. This results in a total of 48 individual α-helices that make up the nanocage as illustrated in Figure 1A. The average energy provided by the calculations for different nanocage structures (sets of the geometric parameters) yielded the structure–energy landscape illustrated in Figure 1B. 

**Figure 2:**
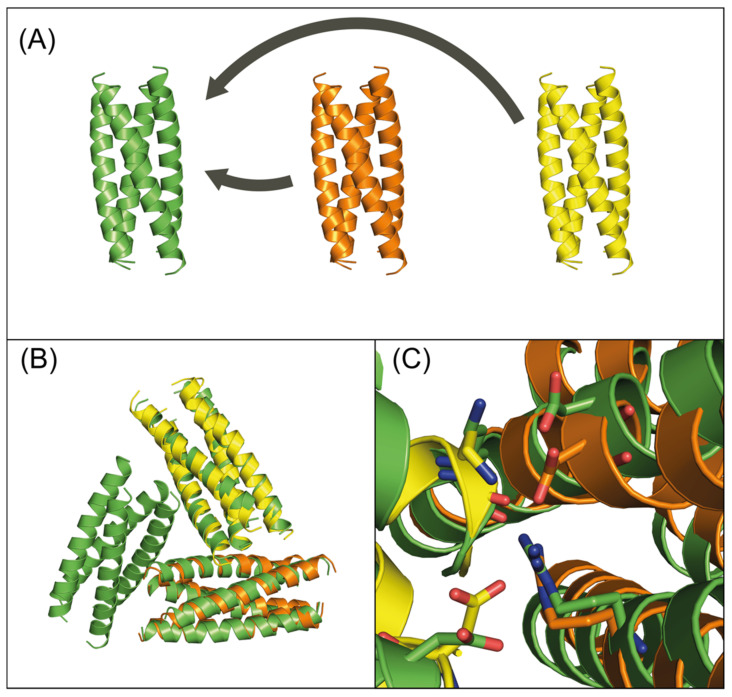
Symmetric 3DCF4 tetramer: (**A**) input structure in green. Yellow and orange subunits are docked against the input by ClusPro; (**B**) overlap of design model and ClusPro prediction. Design model in green, ClusPro prediction in yellow and orange; and (**C**) residue-level view of design model and ClusPro prediction.

The 32 lowest energy minima were subjected to calculations using the sequence symmetry constraint, as well as the full rotamer set. Iterative rounds of protein design were carried out, and the most probable structure of the most probable sequence at each point was subject to further evaluation. The PISA server [54] was used to assess the assembly of each of the predicted cage assemblies. ClusPro [55,56,59] (fast Fourier docking) identified seven candidates in which the alignment between helices in the docked prediction and the model could be visually verified (Figure 2). The selected sequences are given in Table S2.

### 3.2. Experimental Verification

**Figure 3:**
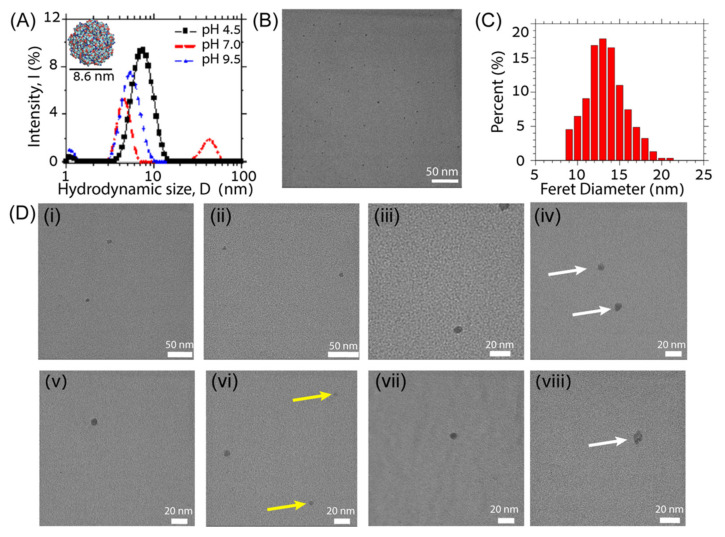
Experimental evidence of coiled-coil nanocage formation: (**A**) dynamic light scattering (DLS) results for 3DCF4 assemblies in different pH solutions showing the presence of coiled-coil cages at pH 4.5; (**B**) wide-view low-resolution transmission electron microscopy (TEM) image showing stable spherical assemblies of 3DCF4 peptide on the grid; (**C**) size distribution of the peptide assemblies indicating particles that are 12–14 nm in diameter; (**D**) high-resolution TEM images of individual coiled-coil cages ca. 8.5 nm in size are shown in (**i**–**viii**). Distorted cages are indicated by white arrows in (**iv**,**viii**) and fragmented cages are indicated by yellow arrows in (**vi**).

Of the seven computationally designed 3D cage-forming (3DCF) peptide sequences that were experimentally investigated, two sequences, i.e., 3DCF4 and 3DCF7, self-assembled into spherical cage-like nanoparticles. Specifically, the candidate sequence 3DCF4 self-assembled into nanocages in dilute peptide solutions (<0.1 mM) and under mild acidic solvent conditions (5 mM acetate buffer, pH 4.5) as evident in both TEM and DLS characterization (Figure 3).

We performed DLS experiments on 3DCF4 peptides assembled in order to quantify the size of 3DCF4 peptide nanocages in their native solution state (Figure 2B). Stable peptide assemblies that were 7.6 nm ± 1.9 nm large were detected in pH 4.5 acetate buffer with a low buffer concentration (5 mM). This hydrodynamic diameter is in close agreement with the expected diameter for an assembled nanocage. The smaller value of the hydrodynamic radius measured here can be attributed to faster diffusion due to the porous and hollow octahedral design of the nanocage. Interestingly, a higher buffer concentration resulted in the fragmentation and aggregation of cage assemblies in solution; this observation is also corroborated by TEM analyses (see Figures S4–S6 in the supplementary information document). The aggregation may be a result of the screening of positive charge on the nanocage by excess buffer ions. Similar fragmentation and aggregation behavior of 3DCF4 peptides was observed under neutral and basic buffer conditions (Figure 3A). The DLS results indicate that a balance of buffer ionic strength and pH that control the net charge of the peptide assembly are crucial for the formation and stabilization of the assembled nanocages in the solution. Similar impacts of multivalent buffer ions on the stability and assembly of coiled coils that carry a net charge have been reported in the literature [60,63].

ImageJ size analysis of multiple wide-view low-magnification cast-film TEM images of 3DCF4 peptides confirms that more than 50% of the spherical assemblies have a Feret diameter of 12–14 nm and form under acidic pH and low ionic strength conditions (Figure 3B,C). The Feret diameter is the longest measured distance between any two points on the boundary of a selected area on the TEM micrograph. Therefore, this overestimates the true size of the assemblies. When the selected areas are fitted to an ellipse, the average major and minor axis lengths are 12.6 nm ± 2.2 nm and 10.4 nm ± 1.9 nm, respectively, which are better estimates of assembly size and closer to the computationally designed value of 9 nm for a properly assembled coiled-coil nanocage. We suspect that the larger particles (14 nm and above) are in aggregates of coiled-coil peptides or dimers or trimers of coiled-coil cages that are difficult to resolve in the TEM images. The larger assembly sizes than the predicted cage size and measured dispersity in sizes may also be a result of the distortion of the hollow cages on the drying and adsorption to the carbon film during cast-film preparation. High-magnification TEM images of successful 3DCF4 assemblies shown in Figure 3D give better estimates of assembly sizes, which yields a ca. 8.5 nm diameter for the spherical assemblies. For more cast-film TEM images, see Figure S3 in supplementary information document.

**Figure 4:**
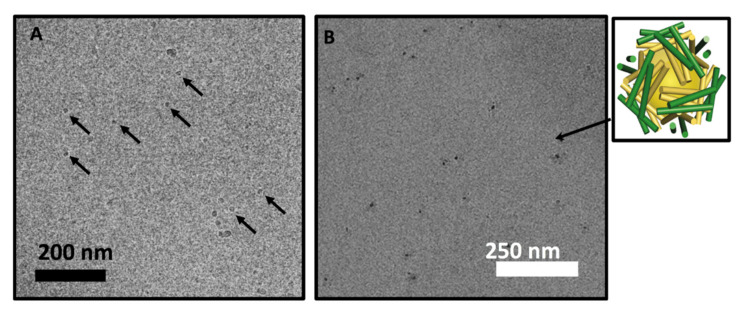
(**A**) Cryo-transmission electron microscopy (cryoTEM) of dialyzed 3DCF4 peptide showing the formation of spherical nanoparticles. Peptides (0.05 mM) were assembled from denaturing conditions (6 M guanidine hydrochloride) to milli-Q water (<6 picomolar guanidine hydrochloride in the final solution); and (**B**) TEM of gold nanoparticles incubated inside 3DCF4 nanocages with an inset showing the schematic of gold incubation within cages.

We further performed Cryo-TEM on 3DCF4 samples that were prepared by a modified method in that the final sample was extensively dialyzed against milli-Q water to remove any residual guanidinium hydrochloride and imaged in unbuffered solution, as can be seen in Figure 4A. The pH of the final solution was slightly acidic (pH 5) as measured using pH paper. Multiple spherical assemblies with homogenous contrast existed with some larger particles that may be dimer and trimer aggregates of nanocages. The average size of assemblies was similar to those measured in cast-film images shown in Figure 3. Furthermore, for demonstrating the utility of the nanocages, we mineralized gold nano-particles within the hollow interior of pre-assembled 3DCF4 nanocages using a simple Au(III) incubation and reduction protocol. Since 3DCF4 nanocages are positively charged under acidic pH conditions, chloroauric ions (AuCl_4_^-^) ions are expected to be initially sequestered within the positively charged hollow interior of the nanocage. The ions are subsequently reduced by borohydride (BH_4_^−^) to nucleate and trigger the growth of gold nanoparticles within the hollow interior of the cage. This method has been used to incubate a host of inorganic particles within viral capsids and apoferritin protein assemblies [10,11]. The cast-film TEM analysis of mineralized 3DCF4 nanocage solution indicated the formation of gold nanoparticles within the cages. Here, encapsulation within the cage is evidenced by the low-contrast peptide halo around the darker gold nanoparticle centers in the cast-film TEM images (Figure 4B). Gold nanoparticles that are 6–8 nm in diameter are readily viewable at low magnifications in cast-film TEM images due to their high contrast (see supplementary information Figure S7 and Table S3 for more information on the size and shape). The formation of some larger gold nanoparticles (>10 nm) and some fused gold nanoparticle assemblies is also evident in the TEM images which allude to the distortion of the peptide assemblies as gold ions nucleate and grow within them, which can subsequently fuse or aggregate. 

## 4. Discussion

We used a computational approach to design a set of α-helical peptide tetramers to assemble into hollow spherical nanocages. The backbone atomic coordinates of the highly stable GCN4-pV coiled coil were chosen as a template on which to design novel sequences enabling intra-bundle interactions, while the bundle core amino acids were retained in order to retain the stability of the tetrameric subunit. However, it is not possible to geometrically construct an octahedral arrangement of coiled-coil subunits without breaking the symmetry between two of the chains in the bundles. To accommodate such an arrangement, we devised a “sequence symmetry” constraint that could be applied to probabilistic sequence optimization calculations. Rotamer probabilities at all sites in the asymmetric unit are calculated independently, but the sum of rotamer probabilities for each amino acid are constrained to be equal at equivalent residue positions. This results in identical sequence profiles for the two chains in the asymmetric unit, but different populations of likely rotamer states. In this manner, we are able to use a homomeric tetramer as the subunit of a highly symmetrical assembly.

We applied the sequence symmetry constraint to the minima of a sequence–structure–energy landscape composed of α-helices in an octahedral arrangement. The resulting models were subjected to fast Fourier transform docking calculations [55,56,57,58,59] to identify subunits that were likely to assemble into the designed structures in the solution. A select number of sequences were chosen for synthesis and experimental characterization. One such sequence, termed 3DCF4, had a solubility in water that made it amenable to solution experiments. The assembly of the cage-forming peptides in the solution was sensitive to experimental conditions, a phenomenon that is well known for protein nanocage formation [64,65]. A high concentration of self-assembling proteins leads to run-away oligomerization, resulting in the formation of aggregates. The same is true of our designed nanocages, which require dilute conditions for formation. In order to prevent the rapid aggregate formation of the peptide subunits, the self-assembly process was carried out in a gradual manner by dialyzing from denaturing conditions to non-denaturing conditions. This process was successful in annealing the system into its target configuration, avoiding kinetic trapping in the local minima. 

Five of the computationally designed sequences could not be assembled into nanocages for various reasons. Firstly, 3DCF1, 3DCF2, and 3DCF3, which were ranked as the most favorable candidates by ClusPro and PISA calculations, proved difficult to synthesize for further investigation. We suspect that this was due to the hydrophobicity of multiple phenylalanine and tryptophan residues in their sequences that caused the aggregation and incomplete formation of the peptides during SPPS cycles. Additionally, for similar reasons, the resulting crude proved difficult to dissolve in water–acetonitrile solvent mixtures and thus purify by RP-HPLC. While 3DCF5 and 3DCF6 were both synthesized and purified, their self-assembly resulted in the formation of non-specific aggregates under all investigated conditions. 

The dialyzed solutions of one specific sequence, i.e., 3DCF4, formed assembled oligomers of well-defined size in the solution with a hydrodynamic diameter consistent with the design model. The particles were directly observed with TEM imaging, further confirming that the spherical design of the nanocages was successful. The other cage forming candidate sequence, 3DCF7 while forming assembled particles under low-ionic strength solution conditions also aggregated into larger clusters under all pH conditions that were experimentally investigated (see supplementary Information, Figure S8). Upon closely comparing the two sequences, one can conclude that both 3DCF4 and 3DCF7 have similar amino acids at all positions except near the N-terminus; the 3DCF7 (pI~7.12) sequence, however, has one extra negatively charged amino acid that shifts its expected isoelectric point closer to neutral pH in comparison to 3DCF4 that has an expected isoelectric point close to 9. This difference evidently has a dramatic effect on the self-assembly of 3DCF7; the presence of multiple oppositely charged amino acids may result in the formation of assemblies that fuse and/or aggregate. 

The close agreement between the particle sizes predicted from the design models and the particle sizes experimentally observed for the 3DCF4 sequence serves to validate the hypothesis that the design methodology is able to identify α-helical peptide sequences with the ability to self-assemble into hollow nanocages capable of cargo encapsulation. 

## 5. Conclusions

The rational and computational design of protein assemblies with hollow interiors remains challenging, as the success rate for accurate assembly designed nanocages is notoriously low [25,66]. In contrast to lower-symmetry complexes, the designed interfaces must be of high fidelity as small defects will propagate and result in aberrant assembly. However, as surface-exposed amino acid side chains are highly dynamic, protein–protein interface design has relied on the use of hydrophobic interactions to drive the association between subunits. A disadvantage of this approach is that the resulting interactions are rather non-specific, so that it is difficult to enforce precise interface geometries by the use of hydrophobic surface patches alone. Tailored hydrogen bonding networks are one promising refinement; the interfaces of designed protein cages have been designed to contain polar interactions that confer structure and improved solubility [66].

A probabilistic approach to protein design enables side-chain conformational variability to be taken into account, facilitating the design of dynamic networks of polar interactions. Additionally, this allows for multi-state design by the use of constraints to identify single sequences which are compatible with multiple local environments. While the designed peptides formed hollow assemblies of the predicted size, the distribution of structures of the resulting particles was not sufficiently uniform to allow for high-resolution structure determination, e.g., by cryo-EM analysis. The designed peptides which were selected as a result of our in silico validation round did in fact tend to contain hydrophobic surface exposed patches, likely as a result of the relatively large weights assigned to hydrophobic interactions in scoring the energetics of protein–protein interfaces. Additional validation steps that filter the initial designs on the basis of hydrogen bond networks and stable polar interactions could be applied to select for assemblies with improved fidelity to the target structure, giving rise to assemblies with highly uniform structures. The use of larger helical bundle subunits could also allow for contacts between subunits that bury larger surface areas, providing further stabilization.

## Data Availability

The data presented in this study are available in supplementary material.

## Supplementary Materials

The following supporting information can be downloaded at: www.mdpi.com/article/10.3390/molecules27041237/s1. Figure S1: Electron spray ionization Mass spectroscopy (ESI-MS) of synthesized and purified peptides: (A) 3DCF4 and (B) 3DCF7 showing the corresponding elution profiles shown in (a), and the ionization i.e. m/z peaks shown in (b). The calculated molecular weights (MW) are 3442 Da for the 3DCF4 peptide and 3448 Da for 3DCF7 peptide; Figure S2: Ellipticity (θ) measured in units of millidegree (mdeg) were measured using circular dichroism and shown in (a) and melting curves shown in (b). Cage-forming peptides were dissolved in 5 mM acetate buffer (pH 4.5) where panel (A) is 3DCF4 and panel (B) is 3DCF7. The negative absorption bands at 208 nm and 222 nm and the positive absorption band at 195 nm are evidence of α-helical secondary structure at room temperature; Figure S3: Cast-film TEM images of 3DCF4 assemblies in low ionic strength acidic solution pH conditions showing formation of stable individual nanoparticles. Scale bar represents 250 nm on micrograph; Figure S4: Circular Dichroism of 3DCF4 peptide dissolved as is in 5 mM acetate buffer (pH 4.5), 5 mM phosphate buffer (pH 7.0) and 5 mM borate buffer (pH 9.5). The negative absorption bands at 208 nm and 222 nm and the positive absorption band at 195 nm are evidence of α-helical secondary structure in this pH range; Figure S5: Dynamic Light Scattering (DLS) data for 3DCF4 assemblies in pH 4.5 acetate buffer at two different buffer concentrations; Figure S6: TEM image of 3DCF4 assemblies in (A) 5 mM phosphate buffer pH 7.0 showing formation of large aggregates of particles (B) 5 mM Borate buffer and (C) 10 mM acetate buffer pH 4.5 showing formation of particles that are consistently smaller in size than expected; Figure S7: Wide-area TEM image of gold incubated nanocages (left) and distribution of Feret diameters in TEM images of the high contrast objects (Au nanoparticles) with mean Feret diameter of 11 ± 2.2 nm; Figure S8: TEM image of 3DCF7 nanocage aggregates at 5 mM phosphate buffer pH 7.0 showing formation of large aggregates of particles; Table S1: Ensemble amino acid type and conformational degrees of freedom. Amino acids and rotamer conformations allowed at each site on the alpha helix; Table S2: Self-assembling peptide candidates. Top seven candidates were ranked by predicted PISA [54] assembly stability score (ΔGint (kcal/mol) provided by PISA) and most probable sequence. Designed residues are colored in red; Table S3: ImageJ Analysis of cages with gold nanoparticles shown in Figure S7.
