# Supplementary material for: Computational Design of Single-Peptide Nanocages with Nanoparticle Templating
Source: Molecules. 2022 Feb 12;27(4):1237. doi: 10.3390/molecules27041237 (PMC8874777; doi:10.3390/molecules27041237)
Supplement: Supplementary file 1 [file molecules-27-01237-s001.zip › molecules-1548660-supplementary.pdf]

S1. Supplementary Information

Computational design

Table S1: Ensemble amino acid type and conformational degrees of freedom. Amino acids and rotamer conformations allowed at each site on the alpha helix.

**Sites**	**Degrees Of Freedom**
1	Acetyl cap – 1 conformation.
31	Amine cap – 1 conformation.
8, 9, 15, 22, 23, 30	Valine – All conformations from Dunbrack 2002 rotamer library.
5, 12, 19, 26, 29	Leucine – All conformations from Dunbrack 2002 rotamer library.
16	Asparigine – 10 most probable conformations from Dunbrack 2002 rotamer library.
2, 3, 4, 6, 7, 10, 11, 13, 14, 17, 18, 20, 21, 24, 25, 27, 28	All amino acids except cysteine and proline considered at each site, using up to the 10 most probable conformations of each amino acid from Dunbrack 2002 rotamer library.

Table S2: Self-assembling peptide candidates. Top seven candidates were ranked by predicted PISA[1] assembly stability score (ΔG_{int} (kcal/mol) provided by PISA) and most probable sequence. Designed residues are colored in red.

Name	*R (Å)*	*θ (°)*	*PISA (kcal/mol)*	*Sequence*
3DCF1	31	44	-1042.4	VIDLTTVVNFLDFVNESLYHVVEWLRVLV
3DCF2	30	46	-986.2	FVDLTTVVNRLDYVNTSLYSVVTWLRVLV
3DCF3	31	54	-907.8	TVDLTHVVWTLDKVNKTLYHVVTLLRILV
3DCF4	30	44	-751.7	EVDLVKVVNRLDTVNKSLYDVVTYLRKLV
3DCF5	32	138	-726.8	EVDLVHVVRDLDYVNKRLYYVVTWLRHLV
3DCF6	31	45	-688.4	DDALVTVVNRLDRVNESLYYVVEDLRKLV
3DCF7	30	43	-680.7	DEDLTRVVNRLDTVNKGLYDVVTYLRKLV

Synthesis and Characterization:

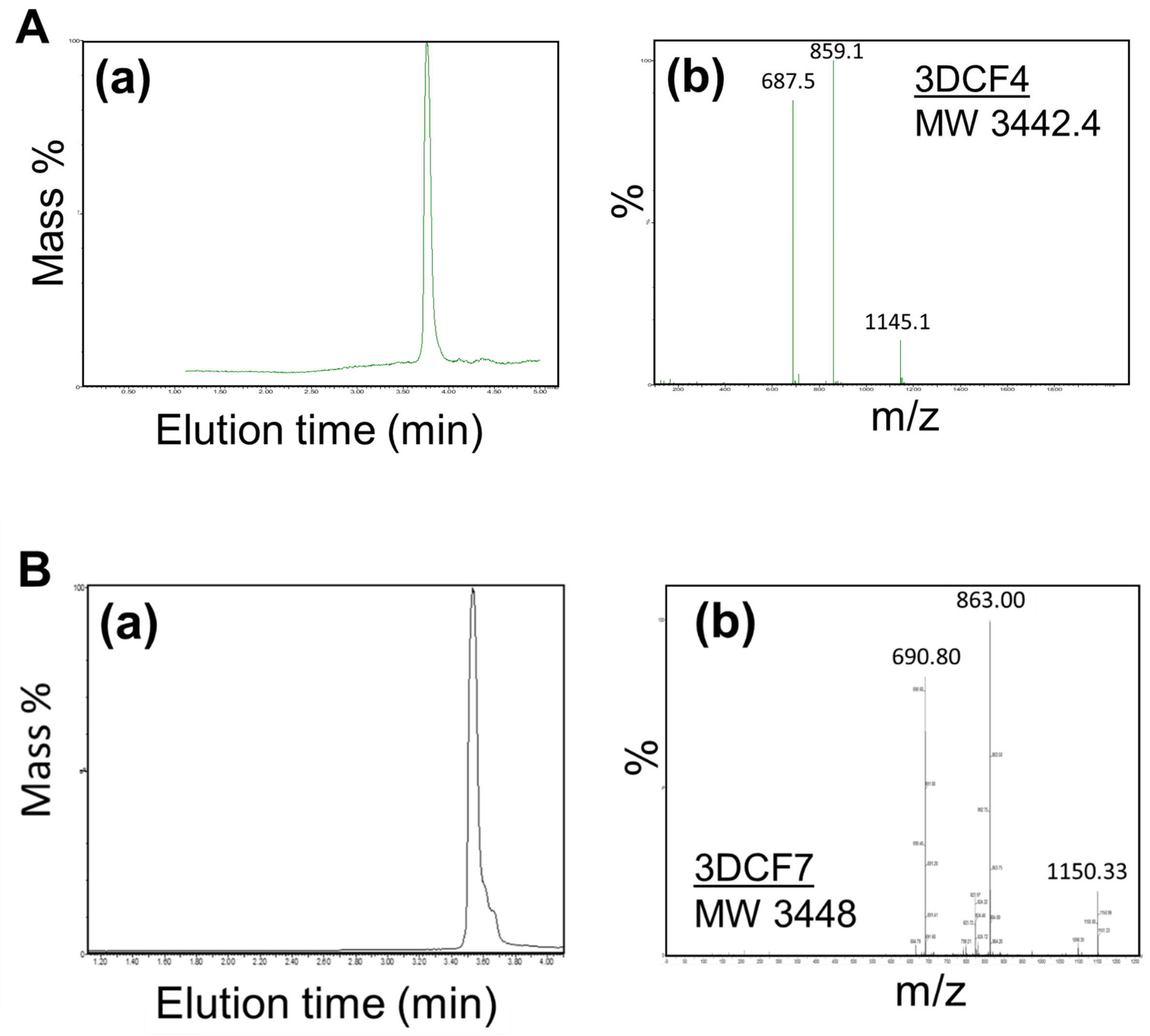

Figure S1: Electron spray ionization Mass spectroscopy (ESI-MS) of synthesized and purified peptides : (A)3DCF4 and (B) 3DCF7 showing the corresponding elution profiles shown in (a), and the ionization i.e. m/z peaks shown in (b). The calculated molecular weights (MW) are 3442 Da for the 3DCF4 peptide and 3448 Da for 3DCF7 peptide.

Circular Dichroic (CD) Spectroscopy

A Jasco J-820 spectropolarimeter (JASCO Inc.) was utilized to investigate the pH dependent secondary structure of the synthesized peptides. 0.1 mM peptide solutions in appropriate buffer were prepared and transferred into an absorption cuvette having a path length of 1 mm (110-QS, Hellma Inc.). Pure buffer solutions with no peptide were used for background subtraction. Sample

spectra in the wavelength range of 200 to 250 nm were recorded at 20°C with a bandwidth of 1 nm and 4 s response time for each data point. The results from three runs were averaged to obtain the final data set. The ellipticity at 208 nm and 222 nm was used to monitor the pH-dependent folding of the peptides into α-helices. The mean residue ellipticity, $[\Theta]_{MRE}$ (deg cm^2 dmol^{-1}), was calculated using the peptide concentration, number of amino acid residues, and cell path length.

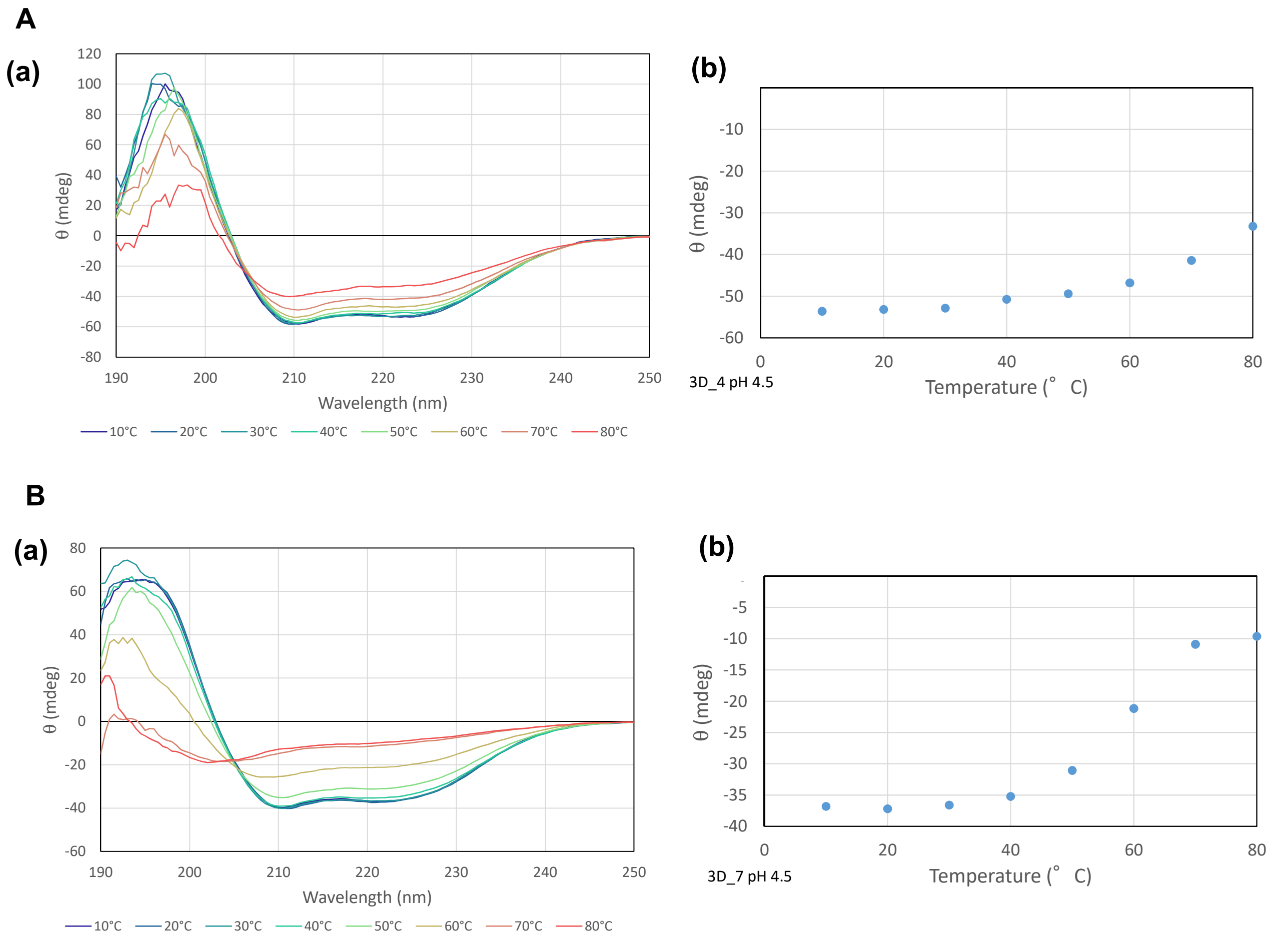

Figure S2: Ellipticity (θ) measured in units of millidegree (mdeg) were measured using circular dichroism and shown in (a) and melting curves shown in (b). Cage-forming peptides were dissolved in 5mM acetate buffer (pH 4.5) where panel (A) is 3DCF4 and panel (B) is 3DCF7. The negative absorption bands at 208 nm and 222 nm and the positive absorption band at 195 nm are evidence of α-helical secondary structure at room temperature.

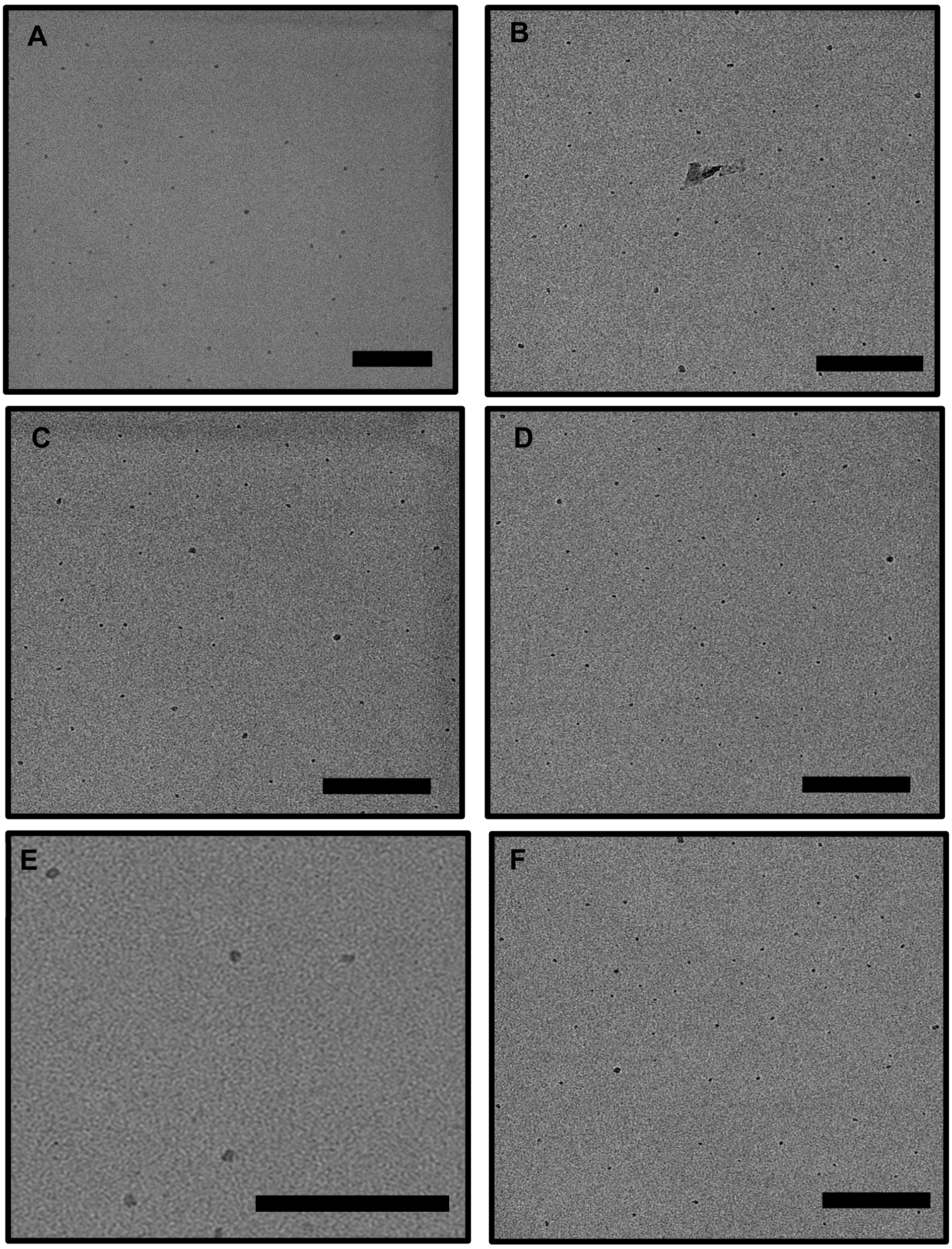

Figure S3: Cast-film TEM images of 3DCF4 assemblies in low ionic strength acidic solution pH conditions showing formation of stable individual nanoparticles. Scale bar represents 250 nm on micrograph.

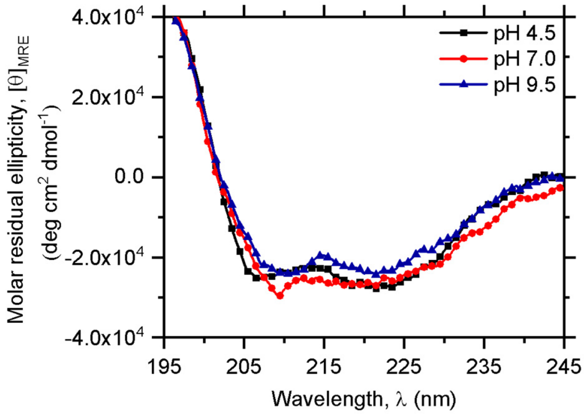

Figure S4: Circular Dichroism of 3DCF4 peptide dissolved as is in 5mM acetate buffer (pH 4.5), 5mM phosphate buffer (pH 7.0) and 5mM borate buffer (pH 9.5). The negative absorption bands at 208 nm and 222 nm and the positive absorption band at 195 nm are evidence of α-helical secondary structure in this pH range.

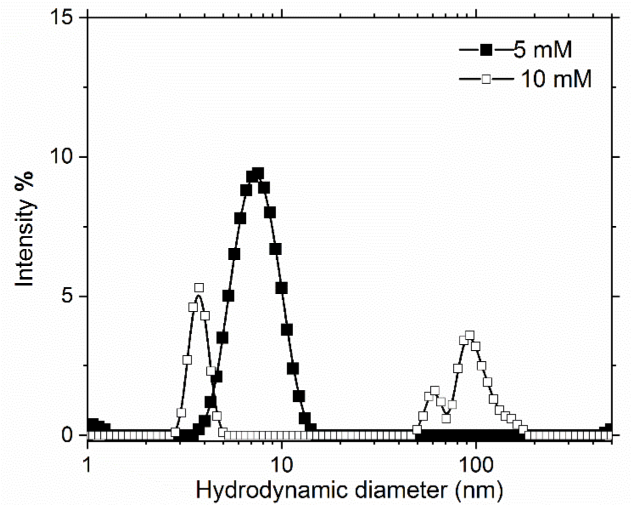

Figure S5: Dynamic Light Scattering (DLS) data for 3DCF4 assemblies in pH 4.5 acetate buffer at two different buffer concentrations.

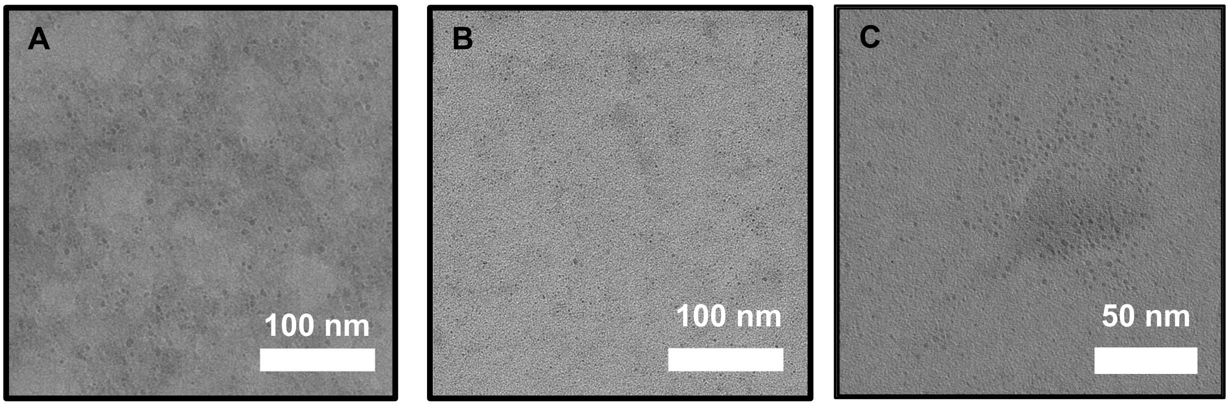

Figure S6: TEM image of 3DCF4 assemblies in (A) 5 mM phosphate buffer pH 7.0 showing formation of large aggregates of particles (B) 5 mM Borate buffer and (C) 10 mM acetate buffer pH 4.5 showing formation of particles that are consistently smaller in size than expected.

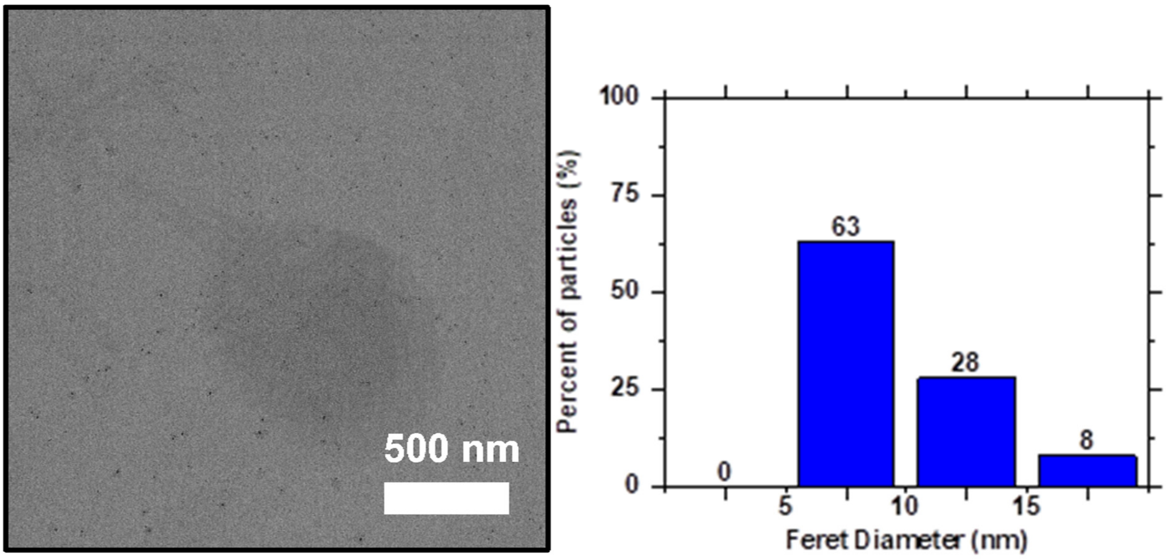

Figure S7: Wide-area TEM image of gold incubated nanocages (left) and distribution of Feret diameters in TEM images of the high contrast objects (Au nanoparticles) with mean Feret diameter of 11 ± 2.2 nm.

Table S3: ImageJ Analysis of cages with gold nanoparticles shown in Fig. S7.

Label	Major axis length (nm)	Minor axis length (nm)	Circularity	Feret diameter (nm)	Aspect Ratio	Solidity
Mean	10.5	5.4	1	11	2	0.9
Standard Deviation	1.6	1.3	0.1	2.2	0.4	0.1
Minimum	9.7	4.1	0.5	9.6	1	0.6
Maximum	19.3	11.2	1	21.6	4.1	1

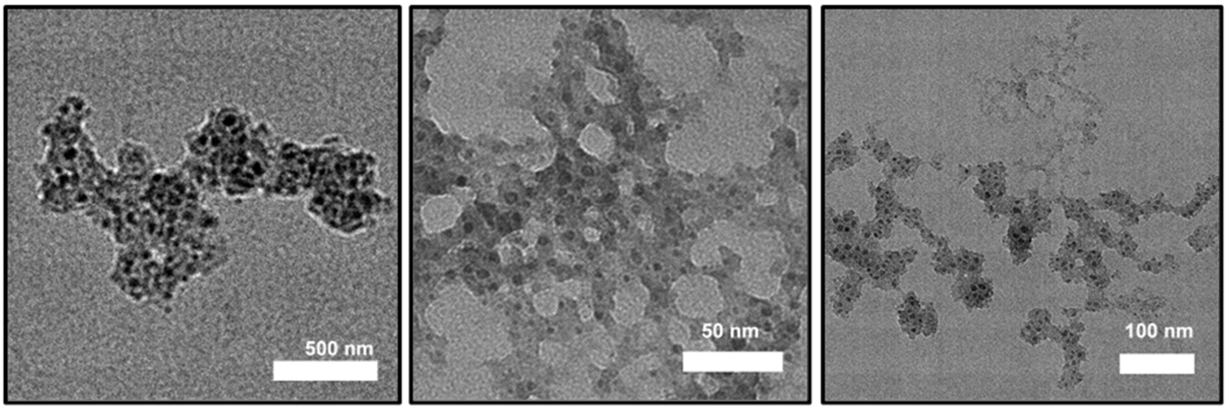

Figure S8: TEM image of 3DCF7 nanocage aggregates at 5 mM phosphate buffer pH 7.0 showing formation of large aggregates of particles.

1. Krissinel, E. & Henrick, K. Inference of Macromolecular Assemblies from Crystalline State. Journal of Molecular Biology vol. 372 774–797 (2007).
